# Fukuyama Congenital Muscular Dystrophy

**DOI:** 10.7759/cureus.21902

**Published:** 2022-02-04

**Authors:** Amit Agarwal, Shyamsunder Sabat, Sangam Kanekar

**Affiliations:** 1 Radiology, Mayo Clinic, Jacksonville, USA; 2 Radiology, University of Florida College of Medicine, Gainesville, USA; 3 Radiology, Penn State University, Hershey, USA

**Keywords:** brain, mri, dystrophy, muscular, congenital

## Abstract

Congenital muscular dystrophy (CMD) is a heterogeneous group of neurological disorders presenting at birth with weakness and hypotonia. Although the diagnosis is finally made through patterns of inheritance and muscle biopsy, the final imaging can be very characteristic in some of the variants, particularly the Fukuyama type of CMD (FCMD). We described the classic imaging findings in a child with this rare condition.

## Introduction

Congenital muscular dystrophies (CMDs) are a heterogeneous group of myopathies with an autosomal recessive inheritance characterized by hypotonia and muscle weakness. These are classified into four different patterns depending upon the clinical, genetic, and radiographic analysis. The four types as classified by Barkovich are Merosin positive (CMD 1), Fukuyama variant (CMD 2), Santavuori muscle-eye-brain (CMD 3), and Walker-Warburg syndrome (CMD 4) [1]. Although the definite diagnosis is made through molecular analysis after muscle biopsy, the magnetic resonance imaging (MRI) findings are classic in vast majority of cases, especially with the Fukuyama variant. However, as these cases are rare, the imaging findings might not be attributed to this specific condition in day-to-day practice. MRI shows distinctive brain anomalies, and a good knowledge of this can be helpful to neuroradiologists and neurologists [1].

## Case presentation

**Figure 1:**
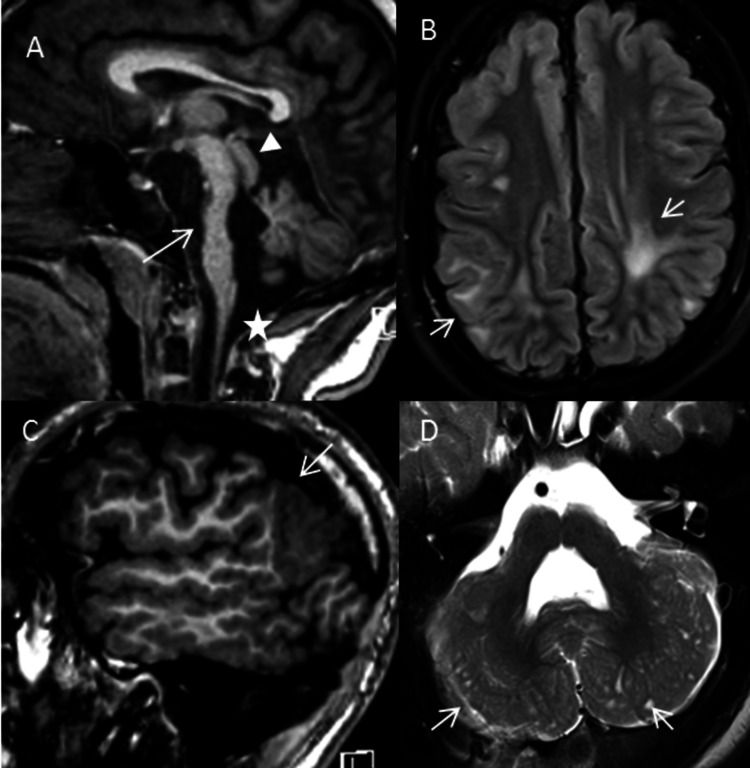
Fukuyama congenital muscular dystrophy (FCMD) A: Sagittal T1 MRI revealing marked hypoplasia of the pons (arrow) with inferior vermian hypoplasia (star) and fused midbrain colliculi (arrowhead). B: Axial FLAIR image showing patchy areas of white matter hyperintensity (arrows). C: Extensive polymicrogyria is noted in the frontal, parietal, and temporal lobes (arrow) on the T1 sagittal image. D: Axial T2 image showing multiple tiny cysts (arrows) adjacent to the dysplastic cerebellar cortex.

A 16-year-old Asian-American male with a diagnosis of Fukuyama congenital muscular dystrophy (FCMD) presented to our hospital for magnetic resonance imaging (MRI) of the brain for worsening seizures and new left-sided retinal detachment. He had been diagnosed with FCMD at the age of three years after presenting with severe developmental delays, feeding problems, and myopic astigmatism. The findings on his muscle biopsy were consistent with dystrophic changes with normal expression of laminin alpha-2 (merosin) and the complete absence of alpha-dystroglycan (a component of muscle absent in true FCMD). As these findings were pathognomonic for FCMD, genetic analysis for underlying fukutin ( FKNT) gene mutation was not felt to be necessary. In the interim, he had multiple office visits and few hospitalizations, predominantly for seizures, vision correction, and feeding issues. Despite diffuse hypotonia and developmental disabilities, he was able to stand without support and follow major commands. MRI of the brain revealed extensive white matter signal abnormalities, marked pontine hypoplasia with hypoplastic inferior vermis, and fused midbrain colliculi. There was diffuse frontotemporal and cerebellar polymicrogyria with multiple small cysts in the lateral cerebellum. The imaging findings were characteristic of FCMD (Figure 1A-1D).

## Discussion

Congenital muscular dystrophy is a heterogeneous group of disorders presenting at birth with weakness, hypotonia, contractures, and dystrophic changes on muscle biopsy. The classification is based on clinical and histopathological findings, along with patterns of inheritance [1]. The clinical presentation is variable, ranging from mild weakness with survival into adulthood to severe form, which is fatal in infancy, primarily due to feeding and respiratory issues. Muscle biopsy is usually performed in all cases of suspected CMD for confirmation and to rule out other causes revealing dystrophic changes in muscle fibers with abnormal fiber size and increase in adipose content and central nuclei size [2]. The four major clinical groups include “pure” CMD with abnormalities limited to the muscles (CMD 1), Fukuyama CMD (CMD 2), muscle-eye-brain disease (CMD 3), and Walker-Warburg syndrome (CMD 4). The most widely accepted imaging classification scheme, suggested by Barkovich, conforms to the clinical classification model [3]. Immunohistochemical tests form the basis for differential diagnosis and identification of specific antibodies for protein deficiencies. Primary or secondary merosin deficiency forms the basis of CMD 1, whereas CMD 2-4 is characterized by dystroglycanopathies. The genes involved in the dystroglycanopathies modify the alpha-dystroglycan protein, which serves as an anchor for the structural framework of the cells in skeletal muscles and plays an important role in the migration of neurons during early stages of development [2]. The “pure” type of CMD is further classified as merosin-positive CMD 1 and merosin-negative CMD 1, both characterized by diffuse hypomyelination with normal cerebral and cerebellar cortex. The clinical findings are mild in merosin-positive variant and moderate to severe in merosin-negative CMD 1 disease. Interestingly, the pure CMD variant may present with minimal to absent neurological signs and symptoms despite the changes detected on MRI. The majority of patients with CMD 2-4 have brain imaging findings that include white matter signal changes, polymicrogyria with or without cystic changes, and varying degrees of posterior fossa malformations including the brainstem and cerebellar hypoplasia [3,4]. There is increasing neurological dysfunction from CMD 2 to CMD 4, with CMD 4 presenting with the most severe brain involvement, both on imaging and on clinical presentation. Fukuyama CMD is characterized by cerebral neocortex and cerebellar cortical polymicrogyria, diffuse central cerebral hypomyelination, and hypoplastic pons and cerebellar vermis. Similar imaging findings are seen with CMD 3 and CMD 4, and demarcation can be challenging on imaging alone. There is significant overlap in clinical features as well with all of these three conditions having ocular anomalies characterized by abnormal eye movements, myopia, and glaucoma. However, Fukuyama CMD has significantly higher tendency to involve the frontal neocortex compared to the other variants, as also seen in our case. Dysplastic corpus callosum and ventriculomegaly are more often seen with CMD 3 and CMD 4, with many cases of CMD showing massive ventricular enlargement [5,6]. Imaging thus serves as an important adjunct to the clinical classification model and can help in understanding the developmental events in these patients [1,3]. Nevertheless, the findings of cortical polymicrogyria, patchy white matter hyperintensities, cerebellar cysts, and brainstem hypoplasia can also be seen with other genetic syndromes such as adhesion G protein-coupled receptor G1 precursor (ADGRG1, previously known as GPR56 mutation) and tubulinopathies (especially TUBBA1A mutation). However, these conditions are comprised of a wide and overlapping range of brain malformations with no involvement of skeletal muscles [7]. Despite similarities in imaging findings, the clinical findings in these conditions are completely different from CMDs. Although Fukuyama CMD is traditionally supposed to be seen in the Japanese population, more and more reports are now emerging of this condition presenting in non-Japanese population. It is therefore important for radiologists to be aware of this entity with the unique constellation of imaging findings [8].

## Conclusions

Congenital muscular dystrophy is a heterogeneous group of disorders with distinctive brain anomalies. Although definite diagnosis is made through clinical and genetic analysis along with muscle biopsy, MRI reveals classic brain anomalies that allow confident diagnosis and also aid in classification into four distinct groups. Using a correct diagnostic algorithm, radiologists can not only diagnose this condition but also help classify it into one of the four subtypes. We describe the characteristic imaging findings in the Fukuyama-type congenital muscular dystrophy with frontal cortical dysplasia, cerebellar polymicrogyria with cysts, and pontine hypoplasia.

## References

[1] Barkovich AJ (1999). Neuroimaging manifestations and classification of congenital muscular dystrophies. AJNR Am J Neuroradiol.

[2] Peat RA, Smith JM, Compton AG (2008). Diagnosis and etiology of congenital muscular dystrophy. Neurology.

[3] Bönnemann CG, Wang CH, Quijano-Roy S (2014). Diagnostic approach to the congenital muscular dystrophies. Neuromuscul Disord.

[4] Leyten QH, Gabreëls FJ, Renier WO, ter Laak HJ (1996). Congenital muscular dystrophy: a review of the literature. Clin Neurol Neurosurg.

[5] van der Knaap MS, Smit LM, Barth PG (1997). Magnetic resonance imaging in classification of congenital muscular dystrophies with brain abnormalities. Ann Neurol.

[6] Aida N, Tamagawa K, Takada K, Yagishita A, Kobayashi N, Chikumaru K, Iwamoto H (1996). Brain MR in Fukuyama congenital muscular dystrophy. AJNR Am J Neuroradiol.

[7] Mutch CA, Poduri A, Sahin M, Barry B, Walsh CA, Barkovich AJ (2016). Disorders of microtubule function in neurons: imaging correlates. AJNR Am J Neuroradiol.

[8] Aida N, Yagishita A, Takada K, Katsumata K (1994). Cerebellar MR in Fukuyama congenital muscular dystrophy: polymicrogyria with cystic lesions. AJNR Am J Neuroradiol.

